# A New Theory of Generation

**Published:** 1882-02

**Authors:** H. D. Valin

**Affiliations:** Member of the Philosophical Society of Chicago, etc.; Chicago


ARTICLE II.

A NEW THEORY OF GENERATION. By H. D. VALIN, M.D., Member of the Philosophical Society of Chicago, etc. (Read before the Biological Society, Jan. 4, 1882.)

"Hippocrates maintains that each of the two sexes possesses two kinds of seeds, formed by the superfluous nutriment, and by fluids constituted of materials proceeding from all parts of the body, and especially from the most essential—the nervous. Of these two seeds, the stronger begets males, the weaker females."*

Buffon supposed organic molecules to be incorruptible primordial monads. After an individual had acquired its full size, organic molecules would be produced in excess in each organ, and they would be sent from it, invested with its shape, to the genital organs.

Darwin declares that every separate part of the whole organization reproduces itself, and that spermatozoa include and consist of a multitude of germs thrown off from each separate part or unit.

All these theories contain certain elements of truth.

Herbert Spencer, in building up the Monistic System of Philosophy, gave the first adequate explanation of generation. "Setting out with physiological units, the existence of which various organic phenomena compel us to recognize, we get an insight into the phenomena of genesis, heredity and variation. If each organism is built of certain highly-plastic units peculiar to its species—units which slowly work toward an equilibrium of their complex polarities, in producing an aggregate of the specific structure, and

* Dunglison's Physiology. Generation.

which are at the same time slowly modifiable by the re-actions of this aggregate—we see why the multiplication of organisms proceeds in the several ways, and with the various results, which naturalists have observed."*

The following pages are intended to explain this process, and show that the theory agrees with the facts, as far as these have been ascertained.

Organized beings grow by an addition of substance, and they perpetuate by division. The most simple process of generation is observed in plants, where a branch coming to touch the ground on account of an increase in weight, shoots new roots and becomes afterward mechanically separated from the parent plant. This process becomes a function in some plants, as in the strawberry (*Frigaria virginiana*), which sends a prolongation of substance which develops at its end into leaves and roots, and becomes separated from the rest by the annual decay of the connecting fiber. This is best seen in the highly developed garden strawberry, and is not present at all in some of the wild varieties, nor in poorly-fed individuals of any variety. A physiological function is evolved from a mechanical process which proved advantageous to the organism thus first affected. The cause of this was that those branches which first touched the ground from an undue increase in substance, took root, and thus generated a new plant in a shorter time than the rest of the branches which went to seed and produced new individuals by seed-growth. The first also imbibed a greater amount of food from the soil, hence the new change was a doubly favorable one, and each branch in which the change occurred had almost triple vitality, triple chances of life, and multiplied so fast as to outstrip the plants which had not been thus first affected. This mode of reproduction is most developed in the banyan-tree of India, a single individual sometimes forming a small forest. In process of time such species of plants entirely cease from multiplying by seed-growths.

Whenever a cloud is blown apart by the wind, its parts continue to increase, while the watery vapor increases in the atmosphere till they condense into rain. If we break in two the first

---

* H. Spencer: Biology, Vol. I, Pt. II, Ch. X.

crystal formed in a crystallizing mixture, both pieces will equally increase by the addition of new crystals. This is a necessary result of gravitation ; and in organic compounds we have the same process. If we cut a worm in two, we make two worms of it, and each one keeps growing. This is the primitive mode of reproduction in the bacteria. After a cell has grown to a greater extent than the instability of its material composition allowed, it breaks into pieces, and the two or more new granules grow into cells, if nutriment is supplied to them.

Upon dividing, the parts of a mass of protoplasm will be most all alike, their further growth will reproduce the parent mass, and their further division will perpetuate the same process of generation by fission. Such primitive mode is observed in the yeast-plant (*Saccharomyces cerevisiœ*).

But it is an axiom that no permanent state of things can endure for any length of time, on account of the perpetual action of gravitation, on unequally distributed matter. Hence an organic granule assumes in its growth certain differentiation ; it becomes a cell by increase, and a chemical change occurs in the cell from an evaporation of water and other constituents ; and by contact with the circumambient medium, a sort of hardening results, and a somewhat different substance forms by the escape of the most unstable atoms of nitrogen, so that this new compound has a more stable chemical affinity. This is a cell-membrane which consists of cellulose ($C_{12}\ H_{20}\ O_{10}$), and under some circumstances resolves itself again into protoplasm.* In fact, after reaching certain organization the mass disintegrates, completely or in part, to resume its first state, and out of each of its granules, which are all alike, other cells will grow, and each one of them will have a tendency to harden on the surface, and thus become invested with a cell-wall or membrane. All masses grow from their centers first, according to the laws of gravitation and chemical affinity, and the first start of the granules is probably the chemical molecule of the compound. By expansion the nucleolus, which does not differ from ordinary organic granules in its appearance, becomes a nucleus, and a nucleus becomes a cell, which is a mass

---

* Bessey's Botany, Ch. III.

of granules; and through such an expansion the nucleus becomes homogeneous as the cell-wall did. In this condition of homogeneity the mass is much more stable, since the less differentiated a substance, the greater its stability or its molecular attraction, which is the same thing.

Any new divisions of this mass of protoplasm result in as many cells, which soon acquire a cell-membrane and a nucleus by further absorption of substance. The first cause of this inheritance of a differentiation on the part of the progeny of these cells is their being placed under similar conditions as the parent, and even if the parent mass had had no cell-membrane nor nucleus, or if it had been deprived of them, the progeny would necessarily form a cell-membrane and a nucleus under the influence of growth and gravitation, and this is proved by reproducing low organisms from an undifferentiated part of their protoplasm. Another cause of such inheritance lies in the fact that when a cell undergoes any differentiation; that is, is affected in some way by any mode of force, each particle of that mass is affected in the same manner to some extent, according to universal attraction, and must show this differentiation more whenever it increases. Thus, any one cell of a higher organism strictly corresponds to the whole, and undergoes the same changes on a small scale; and its growth must, under the same circumstances of force and surroundings, reproduce the parent in every particular. And it will differ from it in the same proportion that the atoms which become added to it, and the modes of force by which it is evolved, differ from the same factors in the parent.

Accordingly, the nearer two cells are to each other, in matters of space, time and composition, the greater their resemblance, and the greater their capacity for reproducing each other, since all modes of force act in inverse ratio to the distance. Thus, after amputation of a limb, the cells of the stump have a tendency to reproduce such a limb, and do so in the lower organisms.

Hence, the reproduction of animals and plants, rightly understood, assumes the character of an exact science, and its results can be mathematically ascertained in advance; and that is precisely what is done to-day by stock-breeders.

Whenever the process of fission is taking place rapidly from an abundance of food, each cell remains in contact, forming a compound organism. A compound of many cells is like a large cell, and necessarily undergoes the same differentiation, on a greater scale than a single cell would; the hardening of its surface becomes an ectoderm instead of a cell-membrane, and the organism grows mostly from the center of the body.

According to the law formulated above, each cell in a compound organism undergoes changes identical with those that the whole undergoes, affected as it is by all the various modes of force; and, if detached from the rest, it will, under the same circumstances, reproduce the animal or plant; so that a part of the mass, or any cell, or any portion of a cell, is absolutely capable of reproducing the parent. Here, natural selection becomes more evident. Those compound organisms which grow rapidly under favorable circumstances will have cells growing faster, maturing earlier, separating from and reproducing the parent organism before the latter has attained its maturity. Here, then, we have a progeny produced during the life of the parent organism, and the animal or plant has a higher degree of viability than the one which ceases to exist in procreating by fission. Hence organisms multiplying by spores, as the above process is called, will survive. They are larger, they multiply more rapidly, and are surely higher than the monads.

A compound organism, upon undergoing more differentiation, ceases to be homogeneous; each of its parts may become compound also, and any one cell will need undergo much differentiation in order to reproduce the parent, somewhat as in growing potatoes or apples from their seeds; and whenever a few of the most differentiated cells aggregate and become separated, or what is still easier, when they separate from the parent and then unite, their further growth will reproduce the organism in a much shorter time, than whenever reproduction takes place through one cell alone. This concrescence of cells is the origin of sexual reproduction. This process does not by any means arise all at once, but it already exists in the monads. The lowest bacteria often mingle together, several coalescing into one, and one dividing into several, though they have undergone no differentiation. And the three processes of generation by fission, by spores and by a concrescence of cells, are sometimes possible in the same animal or plant. In this admixture of cells from the most differentiated parts of an animal or plant, those organisms will grow the faster in which this coalescence is most readily performed. Some of these cells are supposed to arise from the ectoderm, and others from the endoderm, these being the most differentiated parts of monads; at any rate, whenever some of these cells assume a more favorable shape than that of a sphere — and they perpetually change their shape — they penetrate other spherical cells much more readily; hence the early evolution of pointed cells, which are called androspores, and represent the male individual. The same process in the granules of the androspore gives rise to slender, diamond pointed spermatozoa.

In bacteria, the bacillus anthracis shows the evolution of fission into spore-generation, whether it lives in animals, or is cultivated in very rich food, as strongly-oxygenated serum, its growth being seven or eight times more rapid in the latter (*Toussaint*).

In the Gregarinæ we have reproduction by the concrescence of two individuals, and also by simple fission. Iu the Acinata, a species of Infusoria, we meet generation by the concrescence of different individuals; and the two other processes are also possible in these organisms.*

The various grades in the differentiation of spermatozoa from ovules are found in Pandorina, a sort of algæ, in Zygulmaceæ, and in Funaria hygrometrica.†

The spermatozoön is thus more differentiated than the granules of the ovule to which it corresponds, while the ovule itself has its analogue in the seminal vesicle which encloses the spermatozoa, and this explains the physical superiority of the male. However, the ovule, as well as the spermatozoön, and every particle of either, should be adequate to reproduce the entire organism of either sex, and so they are whenever most of the conditions under which the parents were evolved can be gathered in a small compass; but this is only possible in low forms of life.

In the male and female of the human species we have, as it were, two widely differentiated parts of a compound organism,

* Gegenbauer: Comparative Anatomy, §§ 69-70.

† Bessey: Botany.

and the union of the spermatozoön with the ovule brings these elements back to their former homogeneous condition, capable of reproducing either parent. The fact that many more male children are conceived may be ascribed to this higher integration of force in the male, and to an indirect mode of natural selection also ; while the greater mortality of male fœtuses greatly depends on an inferior physical development of the female, as obstetricians will find by noting down the sex of the child in difficult labors. Sexual selection has probably much to do with this also. For, while strength and physical development in the male are great incentives to love from the other sex, the same attributes disparage females in the eyes of the males of the human species.

Any change that an animal undergoes under influences of climate, food, diseases, etc., is also undergone by each of its cells, but specially by the reproductive cells, which do not form part of any differentiated tissue. It could not be otherwise, since all modes of force act at all distances and through all substances. Hence, nothing opposing, the shape, the size, the character, the features, the infirmities, and the entire individuality of the parent will be developed in the progeny. But there are many modes of force opposing this, as the circumstances of time, place and surroundings are changed, the food different, etc. In case the generating substance does not participate in the differentiation of the parent, it must reproduce something more like the ancestors. This is atavism—a common occurrence.

According to the laws of generation, then, peculiar impressions received by the pregnant mother also affect every cell of the fœtus, and that sometimes accounts for those unduly developed faculties, and for idiosyncrasies in the child whose mother has been thus affected during child-bearing. This satisfactorily explains most of what are called cases of maternal impressions, even those extraordinary cases in which the children of a second husband all resemble the first husband. This is the more plausible, when we find that mental impressions are purely dynamical phenomena. This also accounts for a few peculiarities of monsters. This inheritance has been thoroughly demonstrated by the late experiments of M. Mason, of the Royal Academy of Belgium, who succeeded in producing atrophy of the spleen in the progeny of rabbits by removing that organ in the parents. In the same way, the external conditions being attended to, a naturalist has produced all sorts of monsters in chickens, though maternal impressions were impossible here.

If we admit the fact that each animal cell has its nervous system, and all the physiologieal functions, though undeveloped, as explained above, their reaction to all modes of force accounts also for the most extraordinary cases in which children have been known to inherit the vivid perception of facts which their mothers observed while they were carrying them. That is a direct transmission of sensations through the senses of the mother to the brain of the fœtus. Of course, we all know of the inheritance of instincts, as that of birds for building nests, which is a cause of individual existence, as Darwin has shown; and Lindsay, in his valuable work on Comparative Psychology, also demonstrates a transmission of general knowledge, the result of experience in either the father or mother.*

The fact that every cell in the human body is properly an organism, and consequently must be conscious, explains those authenticated cases in which people have received various impressions otherwise than by way of their special senses.

In mentioning the modes of force whose ensemble is called the course of nature, we refer to gravitation, which does not apply only to planets and to inorganic matter, but also to animals, and regulates our every movement; to chemical affinity, by which every substance in the animal body is formed, maintained or renewed; to heat and to electricity, both of which are factors in life; to light, etc., etc.; to natural selection, which plays such a wonderful part in the evolution of animals and plants; and to that redistribution of matter and force on earth by man, at the expense of his own energy, something equivalent to a new creation, and in perpetual opposition to the previous state of things, althóugh its outgrowth. It is also true that a variety of modes of force and their bearings on life and generation, are unknown to us in our present capacity for knowledge.

> "There are more things in heaven and earth, Horatio,

> Than are dreamt of in your philosophy."

* Lindsay: Mind in the Lower Animals, Vol. I.

